# Gut Microbiota and Biomarkers of Endothelial Dysfunction in Cirrhosis

**DOI:** 10.3390/ijms25041988

**Published:** 2024-02-06

**Authors:** Irina Efremova, Roman Maslennikov, Elena Poluektova, Oleg Medvedev, Anna Kudryavtseva, George Krasnov, Maria Fedorova, Filipp Romanikhin, Vyacheslav Bakhitov, Salekh Aliev, Natalia Sedova, Tatiana Kuropatkina, Anastasia Ivanova, Maria Zharkova, Ekaterina Pervushova, Vladimir Ivashkin

**Affiliations:** 1 Department of Internal Medicine, Gastroenterology and Hepatology, Sechenov University, Pogodinskaya Str. 1-1, 119435 Moscow, Russia; ira19940101@yandex.ru (I.E.); polouektova@rambler.ru (E.P.);; 2 Scientific Community for the Promotion of the Clinical Study of the Human Microbiome, Pogodinskaya Str. 1-1, 119435 Moscow, Russia; 3 Consultative and Diagnostic Center 2 of the Moscow Health Department, Millionnaya Str. 6, 107564 Moscow, Russianatalisedova1318@yandex.ru (N.S.); 4 Pharmacology Department, Lomonosov Moscow State University, Leninskie Gori 1, 119991 Moscow, Russia; oleg.omedvedev@gmail.com (O.M.);; 5 Post-Genomic Research Laboratory, Engelhardt Institute of Molecular Biology, Russian Academy of Sciences, Vavilova Str. 32, 119991 Moscow, Russia; 6 First Hospital Surgery Department, Pirogov Russian National Research Medical University, Ostrovityanova Str. 1-7, 117997 Moscow, Russia; 7 Department of Clinical Laboratory Diagnostics, FGBOU DPO “Russian Medical Academy of Continuing Professional Education of the Ministry of Health of the Russian Federation”, Barricadnaya Str. 2/1-2, 125993 Moscow, Russia

**Keywords:** dysbiosis, gut, intestinal permeability, gut–liver axis, gut–heart axis, endothelium

## Abstract

Our aim was to study the association of endothelial dysfunction biomarkers with cirrhosis manifestations, bacterial translocation, and gut microbiota taxa. The fecal microbiome was assessed using 16S rRNA gene sequencing. Plasma levels of nitrite, big endothelin-1, asymmetric dimethylarginine (ADMA), presepsin, and claudin were measured as biomarkers of endothelial dysfunction, bacterial translocation, and intestinal barrier dysfunction. An echocardiography with simultaneous determination of blood pressure and heart rate was performed to evaluate hemodynamic parameters. Presepsin, claudin 3, nitrite, and ADMA levels were higher in cirrhosis patients than in controls. Elevated nitrite levels were associated with high levels of presepsin and claudin 3, the development of hemodynamic circulation, hypoalbuminemia, grade 2–3 ascites, overt hepatic encephalopathy, high mean pulmonary artery pressure, increased abundance of *Proteobacteria* and *Erysipelatoclostridium*, and decreased abundance of *Oscillospiraceae*, *Subdoligranulum*, *Rikenellaceae*, *Acidaminococcaceae*, *Christensenellaceae*, and *Anaerovoracaceae*. Elevated ADMA levels were associated with higher Child–Pugh scores, lower serum sodium levels, hypoalbuminemia, grade 2–3 ascites, milder esophageal varices, overt hepatic encephalopathy, lower mean pulmonary artery pressure, and low abundance of *Erysipelotrichia* and *Erysipelatoclostridiaceae*. High big endothelin-1 levels were associated with high levels of presepsin and sodium, low levels of fibrinogen and cholesterol, hypocoagulation, increased *Bilophila* and *Coprobacillus* abundances, and decreased *Alloprevotella* abundance.

## 1. Introduction

Cirrhosis is the result of chronic liver disease, and its development significantly worsens prognosis [1,2,3,4,5]. The pathological process of cirrhosis is not limited to the liver, but involves other organs and tissues [1,2,3,4,5]. Recently, there has been increased interest in cirrhosis-associated gut pathology, including small intestinal bacterial overgrowth, slowdown of intestinal motility, gut dysbiosis, and an increase in the permeability of the epithelial barrier. Hepatologists have proposed the concept of the gut–liver axis, according to which these intestinal disorders lead to the penetration of intestinal bacteria and their harmful components such as lipopolysaccharide (LPS) from the contents of the intestine into the gut wall, ascitic fluid, liver, and systemic circulation [6,7,8,9,10]. This process is called bacterial translocation [6,11,12,13,14] and leads to the development of local (in the intestinal wall, liver, and blood vessels) and systemic inflammation. The inflammatory environment contributes to the dysfunction of the endothelium, which begins to secrete substances that cause vasodilation (nitric oxide) and vasoconstriction (endothelin-1), and inhibits the formation of vasodilating nitric oxide (asymmetric dimethylarginine [ADMA]) [15,16,17,18]. Dysregulated dilatation of intestinal blood vessels leads to increased splanchnic blood flow, which, together with an increase in ADMA in the portal system resulting in increased vascular resistance to portal blood flow, worsens portal hypertension [15,16,17,18]. This dysregulated splanchnic vasodilation results in a compensatory increase in the work that the heart carries out (hyperdynamic circulation), which further exacerbates the above-mentioned disorders, forming a positive feedback loop [6].

Endothelial dysfunction also plays an important role in the pathogenesis of the pulmonary effects of cirrhosis. A predominant vasodilatory effect leads to the development of hepatopulmonary syndrome, which is caused by a decrease in the relative area of gas exchange (the ratio of the area of gas exchange to the volume of blood flowing through the pulmonary vessels) in excessively dilated pulmonary vessels [19,20,21,22]. In contrast, if vasoconstriction is the predominant effect, portopulmonary hypertension may develop, in which there is an increase in pulmonary vascular resistance and pulmonary arterial hypertension [20,21,22]. Endothelial dysfunction also plays a leading role in the development of hepatorenal syndrome, in which dysfunctional intestinal vasodilation is followed by renal vasoconstriction [23,24,25].

An association of gut microbiota disorders with bacterial translocation [26], systemic inflammation [27,28], and hyperdynamic circulation [28,29] in cirrhosis has been shown, but the associations of gut microbiota taxa with biomarkers of endothelial dysfunction have not been studied. Therefore, the aim of our study was to examine the association of main endothelial dysfunction biomarkers with cirrhosis manifestations, bacterial translocation, and the abundance of gut microbiota taxa.

## 2. Results

**Figure 1:**
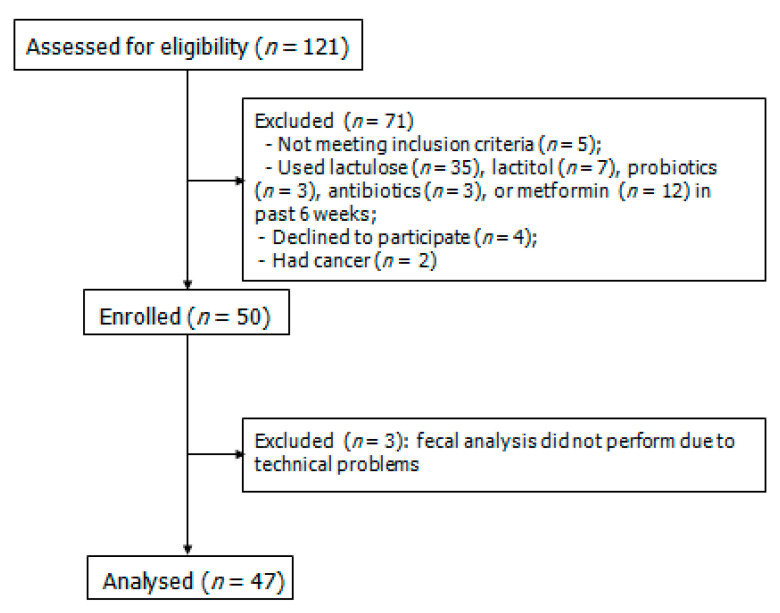
Simplified diagram of the role of the gut–liver axis in the progression of liver fibrosis in chronic liver diseases.

Among the 121 screened patients, 50 met the criteria and were included in the study. Fecal 16S rRNA gene sequencing was not possible in three patients due to technical reasons; these patients were excluded from the study. In total, 47 patients and 27 healthy individuals were assessed (Figure 1). Patients and controls did not differ in age (49 [44–56] vs. 48 [37–56] years; *p* = 0.372) or sex distribution (male/female: 18/29 vs. 10/17; *p* = 0.558). The characteristics of patients with cirrhosis are presented in Supplementary Table S1.

Presepsin, claudin 3, nitrite, and ADMA levels were higher in patients with cirrhosis than in controls. There was no significant difference in plasma big endothelin-1 levels between patients with cirrhosis and controls (Table 1). The presepsin level was significantly correlated (r = 0.322; *p* = 0.027) with the modified dysbiosis ratio ([*Bacilli* (%) + *Proteobacteria* (%)]/[*Clostridia* (%) + *Bacteroidetes* (%)]) [27]. Unfortunately, the levels of LPS in most of the samples were below the minimum threshold for detection. Therefore, it was impossible to assess the levels of this biomarker in our study.

The levels of the biomarkers for endothelial dysfunction were not correlated with each other. The presepsin levels correlated with the levels of nitrites and big endothelin-1 but not with ADMA levels. Claudin 3 levels correlated with nitrite levels but not with the other endothelial dysfunction biomarkers (Table 2).

Plasma nitrite levels correlated with markers of hyperdynamic circulation (increased end-diastolic volume, stroke volume and cardiac output, and reduced systemic vascular resistance) and mean pulmonary artery pressure. Other biomarkers for endothelial dysfunction did not significantly correlate with the markers of hyperdynamic circulation in cirrhosis. Serum albumin levels were inversely correlated with nitrite and ADMA levels, fibrinogen levels were inversely correlated with big endothelin-1 levels, cholesterol levels were inversely correlated with nitrite and big endothelin-1 levels, and serum sodium levels were directly correlated with big endothelin-1 levels and inversely correlated with ADMA levels. The grade of esophageal varices was inversely related to ADMA levels. Of all the biomarkers for endothelial dysfunction, only the plasma levels of ADMA were significantly correlated with the severity of cirrhosis according to the Child–Pugh score. There were no other significant correlations between endothelial dysfunction biomarkers and the main parameters of cirrhosis (Table 3).

Patients with grade 2–3 ascites had higher nitrite and ADMA levels than patients without this manifestation of cirrhosis. This was also true for overt hepatic encephalopathy and hypoalbuminemia. Patients with grades 2–3 esophageal varices had lower ADMA levels than patients with grades 0–1 esophageal varices. Hypocoagulation was associated with higher levels of big endothelin-1. Patients with portopulmonary hypertension had higher nitrite levels and lower ADMA levels than patients without this manifestation of cirrhosis. At the same time, the presence of covert hepatic encephalopathy and hyperbilirubinemia was not associated with any changes in the level of the studied endothelial dysfunction biomarkers in cirrhosis (Table 4).

The plasma nitrite levels were inversely correlated with values of the Chao1 (r = −0.287; *p* = 0.050) and ACE (r = −0.287; *p* = 0.050) gut microbiota biodiversity indices, but not with the Shannon index (*p* = 0.076). The plasma ADMA and big endothelin-1 levels were not significantly correlated with these gut microbiota biodiversity indices (*p* > 0.050).

**Figure 2:**
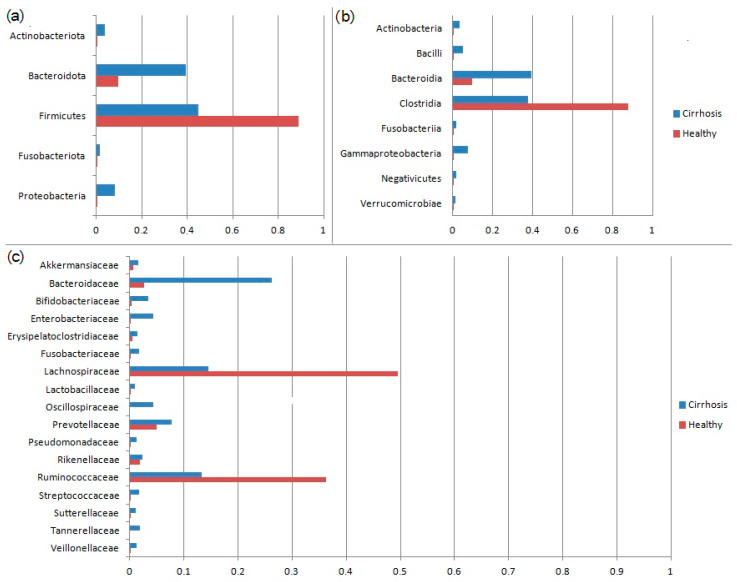
The gut microbiota of the patients with cirrhosis and healthy controls at the level of main (with average relative abundance above 1%) phyla (**a**), classes (**b**), and families (**c**).

Among gut microbiota taxa (Figure 2), the plasma nitrite levels were directly correlated with the abundance of *Proteobacteria*, *Alcaligenaceae*, *Comamonadaceae*, *Enterobacteriaceae*, *Erysipelatoclostridium*, and *Escherichia-Shigella*, and inversely correlated with the abundance of *Anaerovoracaceae*, *Brevinemataceae*, *Oscillospiraceae*, *Peptococcaceae*, *Rikenellaceae*, *Anaerotruncus*, *Brevinema*, *Paludicola*, *Papillibacter*, and *Peptococcus*. ADMA levels were directly correlated with *Lactococcus* abundance and inversely correlated with the abundance of *Erysipelotrichia*, *Erysipelatoclostridiaceae*, and *Fournierella*. Big endothelin-1 levels were directly correlated with *Bilophila* and *Sutterella* abundance and inversely correlated with the abundance of *Alcaligenaceae*, *Caulobacteraceae*, *Achromobacter*, *Alloprevotella*, *and Caulobacter* (Table 5).

Patients with high nitrite levels (fourth quartile) had an increased abundance of *Proteobacteria* and *Erysipelatoclostridium* and a reduced abundance of *Oscillospiraceae*, *Subdoligranulum*, *Rikenellaceae*, *Acidaminococcaceae*, *Christensenellaceae*, *Anaerovoracaceae*, *Peptococcaceae*, *Anaerotruncus*, and *Paludicola* in the gut microbiota compared to those with low nitrite levels (first quartile). Patients with high ADMA levels (fourth quartile) had an increased abundance of *Bilophila*, *Lactococcus*, and *Dielma* and a reduced abundance of *Erysipelotrichia*, *Erysipelatoclostridiaceae*, and *Fournierella* in the gut microbiota compared to those with low AMDA levels (first quartile). Patients with high big endothelin-1 levels (fourth quartile) showed an increased abundance of *Bilophila* and *Coprobacillus* and a reduced abundance of *Alloprevotella*, *Pedobacter*, and *Sphingobacteriaceae* in the gut microbiota compared to those with low big endothelin-1 levels (first quartile) (Table 6).

## 3. Discussion

The aim of our study was to investigate the missing link in the gut–liver axis by evaluating the association of biomarkers for endothelial dysfunction with gut microbiota taxa as well as with manifestations of cirrhosis [6].

Nitric oxide is extremely unstable and very quickly converts into nitrite ions, the measurement of which in blood plasma can be considered a substitute for the determination of the level of this major endothelial vasodilator [30]. In our study, it was shown, as expected, that a high nitrite level was associated with systemic vasodilation and hyperdynamic circulation. These pathological conditions contributed to the worsening of portal hypertension, which resulted in the association of high blood nitrite levels with more severe ascites. Albumin sequestration in ascitic fluid could be the cause of the association between high nitrite levels and hypoalbuminemia. At the same time, the presence of minimal ascites did not depend on the levels of endothelial dysfunction biomarkers, which suggests that endothelial dysfunction may be a “second hit” in the pathogenesis of this disorder. Increased circulating blood volume as a compensatory response to arterial vasodilation induced by nitric oxide leads to an increase in the amount of blood in the vessels of the lungs and the development of pulmonary hypertension. These results are consistent with published data [30,31,32].

It has previously been shown that the levels of nitrites in cirrhosis are directly correlated with the levels of LPS in the blood [30], which is considered to be an indicator of bacterial translocation [33]. Unfortunately, there is no “gold standard” biomarker for bacterial translocation [33]. LPS is a direct marker of bacterial invasion; however, it is only present in Gram-negative bacteria, and cannot therefore be used to detect bacterial translocation of Gram-positive bacteria. Presepsin, a fragment of the CD14 molecule which is involved in the recognition of conserved pathological patterns in Gram-positive and Gram-negative bacteria and their phagocytosis, may be a more suitable universal biomarker for bacterial translocation [33]. Presepsin is formed as a result of CD14 proteolysis in the phagolysosome and is released by phagocytes into the external environment. The treatment of phagocytes with LPS did not lead to the formation of presepsin, in contrast to treatment with live or dead bacterial cells. This protein is considered to be a diagnostic biomarker for sepsis [34,35,36,37,38]. Therefore, presepsin can be considered as a universal biomarker for the cellular bacterial translocation of both Gram-positive and Gram-negative bacteria, while LPS is a biomarker for both the cellular translocation of whole living or dead Gram-negative bacterial cells and the molecular translocation of single LPS molecules or LPS molecules in fragments of dead Gram-negative bacteria. Presepsin has only recently begun to be studied in patients with cirrhosis [39,40,41,42,43]. Our study showed a positive correlation between presepsin levels and the severity of dysbiosis, providing further evidence of its suitability as a biomarker for bacterial translocation.

Our study showed a direct correlation between the levels of presepsin and the levels of nitrites and big endothelin-1, which suggests that these biomarkers of endothelial dysfunction depend on the extent of bacterial translocation.

In addition, the levels of nitrites were directly correlated with claudin 3 levels, a biomarker for intestinal barrier dysfunction that leads to increased intestinal permeability. This protein is a component of the tight junctions of the intestinal epithelium, and its level increases following endothelial damage, when the permeability of the intestinal barrier is increased [44,45]. Zonulin, which is considered as a biomarker for increased intestinal permeability, is a protein regulator of this permeability rather than a direct result of this process [46,47,48]. Claudin 3 has previously been correlated with biomarkers of both molecular bacterial translocation (LPS) and systemic inflammation [44]. In our study, the level of claudin 3 was significantly correlated with the level of nitrites, but not with the other biomarkers of endothelial dysfunction. This may be because nitric oxide production is affected by both molecular (LPS) and cellular bacterial translocation, and the production of big endothelin-1 is only affected by cellular bacterial translocation. ADMA levels do not only depend on global bacterial translocation but can be determined by the effect of individual bacterial taxa.

Among the taxa of the gut microbiota associated with high levels of nitrites, *Proteobacteria* have special significance. This phylum is mainly represented in the gut microbiota by harmful bacteria with active LPS. An increase in the abundance of these microbes is a typical manifestation of cirrhosis-associated gut dysbiosis [26,27,49,50,51]. Therefore, it can be assumed that an increase in the number of LPS-forming Proteobacteria and an increase in the permeability of the intestinal barrier contribute to increased nitric oxide production in intestinal blood vessels, leading to splanchnic vasodilation and aggravation of the above pathological processes.

The roles of other taxa of the gut microbiota were associated with an increase in the blood nitrite level in cirrhosis in our study remain to be established.

**Figure 3:**
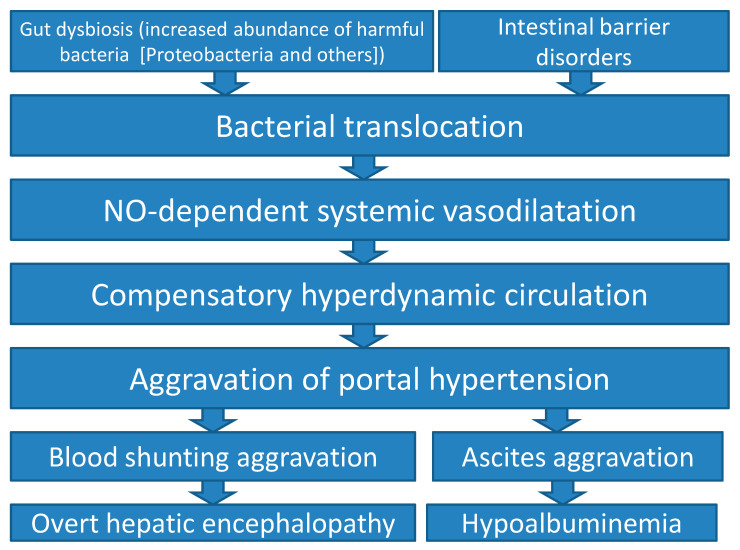
Simplified scheme of disorders of the gut–endothelium–heart–liver axis in cirrhosis according to our study and literature data.

Therefore, based on our data and that from the literature, the following simplified scheme of disorders of the gut-endothelium-heart-liver axis in cirrhosis can be proposed (Figure 3).

ADMA is an endothelial-mediated inhibitor of nitric oxide formation and can be considered an indirect vasoconstrictor [52,53,54,55,56,57]. It is thought to substantially contribute to the narrowing of portal veins, which increases portal blood flow resistance and contributes to the development of portal hypertension [15,18,57,58]. It has previously been shown that the level of this biomarker in the blood increases with the severity of cirrhosis and does not significantly correlate with the level of nitrites in decompensated cirrhosis [57,58,59], which is consistent with our data. In the present study, high ADMA levels were associated with the presence of grade 2–3 ascites, as well as with hypoalbuminemia and hyponatremia associated with the sequestration of these substances in ascitic fluid. Interestingly, in contrast to a previously published study [60], we observed an inverse relationship between serum ADMA levels and the degree of esophageal varices. This can be explained by the venoconstrictive effect of this molecule. Further studies are required to clarify this result.

ADMA was the only endothelial dysfunction biomarker that was not significantly correlated with either the biomarkers of bacterial translocation or the biomarkers of intestinal barrier dysfunction. However, an inverse correlation was observed between blood ADMA levels and the abundance of *Erysipelotrichia* in the gut microbiota. It is possible that the relationship between these bacteria and this biomarker of endothelial dysfunction is not directly related to bacterial translocation; this will need to be established by future research.

Big endothelin-1 is a more stable precursor of endothelin-1 [61], which is the main endothelium-dependent vasoconstrictor [62,63,64]. High levels of endothelin-1 are associated with the development of hepatopulmonary syndrome [65]. It is assumed that endothelin-1 stimulates the endothelium of the pulmonary vessels to intensively produce nitric oxide, thereby providing a paradoxical vasodilating effect in this case [19,20]. There were no patients with such a complication of cirrhosis in our study. High levels of endothelin-1 are also associated with the development of portopulmonary hypertension in cirrhosis [66,67,68,69]. This biomarker of endothelial dysfunction was the only one not significantly elevated in cirrhosis, possibly indicating a minor role for this molecule in the pathogenesis of this disease. Its levels were not correlated with any of the main manifestations of cirrhosis, with the exception of hypocoagulation. It is most likely that this correlation, as well as correlations with sodium and fibrinogen levels, is general and not limited to cirrhosis. However, its level increased with the level of the cell bacterium translocation biomarker presepsin and was associated with changes in the abundance of a number of minor taxa in the gut microbiome. This does not allow us to rule out its participation in the gut–liver axis in cirrhosis; further studies are required to clarify the position of this molecule. Moreover, earlier studies showed that endothelin-1 levels are higher in cirrhotic patients than in healthy individuals and increase with increasing severity of cirrhosis [69,70,71,72,73]. However, other studies did not show a significant difference in the endothelin-1 levels between cirrhotic patients and healthy individuals [74,75], which matches our data. Several authors, although they found an increased level of endothelin-1 in the blood of patients with cirrhosis, did not reveal its relationship with the severity of this disease [76]. Perhaps this is due to the fact that the production of endothelin-1, which is reflected by the level of big endothelin-1, did not increase significantly in cirrhosis in contrast with its conversion into the active form. Further research with simultaneous studies of the levels of endothelin-1 and big endothelin-1 in patients with cirrhosis are required to clarify the place of this molecule in the gut–liver axis.

The strength of our study is that it is the first to evaluate the association of various biomarkers of endothelial dysfunction with gut microbiota taxa, a proposed biomarker of bacterial cell translocation, and a proposed direct biomarker of intestinal barrier dysfunction in cirrhosis. In addition, we differentially investigated the relationship between a group of principal endothelial dysfunction biomarkers and the main manifestations of cirrhosis. The limitation of our study is the small number of patients, which nevertheless allowed us to obtain significant results. 

## 4. Material and Methods

### 4.1. Patients

Patients with cirrhosis who presented to the Department of Hepatology’s Clinic for Internal Diseases, Gastroenterology, and Hepatology for routine examination were screened for participation in this observational study. The study procedures were explained to the potential participants, and written informed consent was obtained before enrollment. The study was approved by the Local Ethics Committee (#03-16) and performed in accordance with the Declaration of Helsinki.

The inclusion criteria were the presence of cirrhosis, the diagnosis of which was made based on histology or a combination of physical examination, laboratory and instrumental data, signed written informed consent, and an age of between 18 and 70 years. The exclusion criteria were as follows: use of drugs that could affect the composition of the gut microbiota (lactulose, lactitol, or other prebiotics, probiotics, antibiotics, and metformin) in the preceding six weeks; alcohol consumption in the preceding six weeks; current infection (except spontaneous bacterial peritonitis); inflammatory bowel disease, cancer, renal failure, or any other serious disease.

The control group consisted of 27 healthy individuals who visited the clinic for routine health examinations during the same period.

### 4.2. Investigations

The day after the initial medical examination, fasting blood was collected from patients and immediately centrifuged. The plasma was separated, divided into several aliquots, and frozen. Once all patients were recruited, aliquots were thawed and the levels of biomarkers for endothelial dysfunction (big endothelin-1, nitrites [nitrates were also detected by this method, but we will use the term “nitrites” to simplify the text], and ADMA), intestinal barrier dysfunction (claudin 3 [44]), and bacterial translocation (presepsin and LPS) were measured. Big endothelin-1 (Big-ET-1 kit; BlueGene Biotech, Shanghai, China), ADMA (catalog no. CEB301Ge; Cloud-Clone Corp., Katy, TX, USA), claudin 3 (catalog no. SEF293Hu; Cloud-Clone Corp.), LPS (catalog no. SEB526Ge; Cloud-Clone Corp.), and presepsin (catalog no. S018-sCD14; Cloud-Clone Corp.) levels were assessed by enzyme immunoassay. Nitrite levels were examined by photometry (catalog no. A013-2; Cloud-Clone Corp.). Assays were performed according to the manufacturers’ instructions. The blood plasma of healthy controls was examined in the same way.

Stool samples were collected from patients on the same day as blood, and immediately frozen for 16S rRNA gene sequencing as described previously [27]. The average number of reads per sample was 54,050, with a minimum of 6695. The number of unassigned reads when analyzing phyla and classes of gut microbiota was less than 1%; when assessing families, it was 3.2%; and when assessing genera, it was 14.3%.

The following day, patient systemic hemodynamics were assessed using echocardiography with simultaneous measurement of blood pressure and heart rate by the oscillometric method, as described previously [28].

All patients underwent a standard examination that included physical and neurological examination, abdominal ultrasound, complete blood count, blood chemistry, coagulation tests, and a number connection test for the diagnosis of covert hepatic encephalopathy.

### 4.3. Statistical Analysis

Statistical analysis was performed with STATISTICA 10 (StatSoft Inc., Tulsa, OK, USA) software. The data are presented as medians [interquartile ranges]. Differences between continuous variables were assessed with the Mann–Whitney method. Fisher’s exact test was used to assess the differences between categorical variables. Correlations between variables were computed using Spearman’s rank test. *p*-values ≤ 0.05 were considered to be statistically significant. Significant differences are marked in bold and italics in the tables.

## 5. Conclusions

Gut microbiota taxa and bacterial translocation are differently associated with endothelial dysfunction biomarkers, which are variously associated with manifestations of cirrhosis.

## Figures and Tables

**Table 1:** Plasma levels of biomarkers in patients with cirrhosis and healthy controls.

Biomarker	Cirrhosis	Controls	*p*
***Nitrite, µmol/L***	***120 [32–161]***	***63 [43–106]***	***0.026***
***ADMA, ng/mL***	***109 [93–132]***	***93 [90–100]***	***0.006***
Big endothelin-1, nmol/mL	1.4 [0.7–3.1]	1.5 [1.0–2.0]	0.821
***Claudin 3, ng/mL***	***11.9 [8.5–16.5]***	***9.3 [8.5–11.3]***	***0.032***
***Presepsin, ng/mL***	***0.43 [0.09–1.98]***	***0.12 [0.10–0.14]***	***0.011***

**Table 2:** Correlation matrix of biomarkers for endothelial dysfunction, bacterial translocation, and increased intestinal permeability in patients with cirrhosis.

Biomarker	Nitrite	ADMA	Big Endothelin-1	Presepsin	Claudin 3
Nitrite	-	NS	NS	r = 0.310; *p* = 0.034	r = 0.295; *p* = 0.044
ADMA	NS	-	NS	NS	NS
Big endothelin-1	NS	NS	-	r = 0.313; *p* = 0.039	NS
Presepsin	r = 0.310; *p* = 0.034	NS	r = 0.313; *p* = 0.039	-	NS
Claudin 3	r = 0.295; *p* = 0.044	NS	NS	NS	-

NS—Not significant.

**Table 3:** Correlations of endothelial dysfunction biomarkers with parameters of systemic hemodynamics and main indicators in patients with cirrhosis.

	Nitrite	ADMA	Big Endothelin-1
Age	NS	NS	NS
Body mass index	NS	NS	NS
Child–Pugh score	NS	r = 0.396; *p* = 0.006	NS
End-diastolic volume of the left ventricle	r = 0.301; *p* = 0.040	NS	NS
Ejection fraction of the left ventricle	NS	NS	NS
Stroke volume	r = 0.369; *p* = 0.011	NS	NS
Heart rate	NS	NS	NS
Cardiac output	r = 0.334; *p* = 0.022	NS	NS
Mean blood pressure	NS	NS	NS
Systemic vascular resistance	r = −0.330; *p* = 0.024	NS	NS
Mean pulmonary artery pressure	r = 0.298; *p* = 0.042	NS	NS
Esophageal varices grade	NS	r = −0.414; *p* = 0.004	NS
Total serum protein	NS	NS	NS
Serum albumin	r = −0.289; *p* = 0.049	r = −0.367; *p* = 0.011	NS
Total serum bilirubin	NS	NS	NS
International normalized ratio	NS	NS	NS
Fibrinogen	NS	NS	r = −0.476; *p* = 0.001
Serum creatinine	NS	NS	NS
Serum sodium	NS	r = −0.291; *p* = 0.047	r = 0.400; *p* = 0.007
Serum potassium	NS	NS	NS
Serum glucose	NS	NS	NS
Serum cholesterol	r = −0.358; *p* = 0.013	NS	r = −0.378; *p* = 0.011
Alanine aminotransferase	NS	NS	NS
Aspartate aminotransferase	NS	NS	NS
C-reactive protein	NS	NS	NS
Splenic length	NS	NS	NS

NS—not significant.

**Table 4:** Endothelial dysfunction biomarkers and manifestations of cirrhosis.

Biomarker	Manifestation Present	Manifestation Absent	*p*
Ascites			
Nitrite, µmol/L	120 [30–161]	128 [73–158]	0.792
ADMA, ng/mL	109 [96–129]	111 [84–145]	0.774
Big endothelin-1, nmol/mL	1.42 [0.91–3.07]	1.23 [0.68–3.07]	0.576
Grades 2–3 ascites			
***Nitrite, µmol/L***	***149 [120–186]***	***92 [19–154]***	***0.010***
***ADMA, ng/mL***	***129 [114–159]***	***104 [90–120]***	***0.042***
Big endothelin-1, nmol/ml	1.69 [1.22–3.07]	1.31 [0.68–3.07]	0.275
Grades 2–3 esophageal varices			
Nitrite, µmol/L	105 [30–155]	129 [69–164]	0.462
***ADMA, ng/mL***	***100 [84–114]***	***126 [107–164]***	***0.006***
Big endothelin-1, nmol/ml	1.48 [0.75–3.07]	1.41 [0.68–3.07]	0.676
Covert hepatic encephalopathy			
Nitrite, µmol/L	110 [39–150]	141 [9–168]	0.837
ADMA, ng/mL	109 [90–128]	100 [84–114]	0.550
Big endothelin-1, nmol/mL	1.45 [0.75–2.96]	0.91 [0.54–3.29]	0.426
Overt hepatic encephalopathy			
***Nitrite, µmol/L***	***179 [166–187]***	***114 [30–153]***	***0.009***
***ADMA, ng/mL***	***139 [129–170]***	***107 [88–124]***	***0.038***
Big endothelin-1, nmol/mL	2.51 [1.51–4.06]	1.37 [0.70–3.02]	0.698
Hypoalbuminemia (serum albumin < 35 g/L)			
***Nitrite, µmol/L***	***149 [114–172]***	***60 [5–145]***	***0.003***
***ADMA, ng/mL***	***118 [103–151]***	***99 [77–123]***	***0.017***
Big endothelin-1, nmol/mL	1.45 [1.11–3.07]	1.25 [0.68–3.07]	0.320
Hypocoagulation (international normalized ratio > 1.7)			
Nitrite, µmol/L	129 [78–161]	119 [32–155]	0.581
ADMA, ng/mL	126 [100–150]	109 [88–124]	0.194
***Big endothelin-1, nmol/mL***	***3.10 [1.41–3.84]***	***1.22 [0.71–2.39]***	***0.044***
Hyperbilirubinemia (serum total bilirubin > 34 µmol/L)			
Nitrite, µmol/L	121 [55–159]	119 [4–169]	0.656
ADMA, ng/mL	111 [98–139]	100 [84–129]	0.438
Big endothelin-1, nmol/mL	1.42 [091–3.07]	1.33 [0.71–3.29]	0.787
Portopulmonary hypertension (mean pulmonary artery pressure ≥ 25 mm Hg)			
***Nitrite, µmol/L***	***149 [119–172]***	***92 [1–151]***	***0.035***
***ADMA, ng/mL***	***96 [75–117]***	***114 [100–147]***	***0.048***
Big endothelin-1, nmol/ml	1.8 [0.7–3.3]	1.4 [0.7–3.0]	0.504

**Table 5:** Significant correlations between plasma levels of endothelial dysfunction biomarkers and gut microbiota taxa.

Direct Correlations			Inverse Correlations		
Taxon Rank	Taxon	Correlation Coefficient; *p*-Value	Taxon Rank	Taxon	Correlation Coefficient; *p*-Value
Nitrite					
Phylum	*Proteobacteria*	0.327; 0.025	Family	*Anaerovoracaceae*	−0.363; 0.012
Family	*Alcaligenaceae*	0.298; 0.043	Family	*Brevinemataceae*	−0.324; 0.026
Family	*Comamonadaceae*	0.317; 0.030	Family	*Oscillospiraceae*	−0.295; 0.044
Family	*Enterobacteriaceae*	0.293; 0.046	Family	*Peptococcaceae*	−0.383; 0.008
Genus	*Erysipelatoclostridium*	0.288; 0.049	Family	*Rikenellaceae*	−0.307; 0.036
Genus	*Escherichia-Shigella*	0.295; 0.044	Genus	*Anaerotruncus*	−0.337; 0.021
			Genus	*Brevinema*	−0.324; 0.027
			Genus	*Paludicola*	−0.357; 0.014
			Genus	*Papillibacter*	−0.460; 0.001
			Genus	*Peptococcus*	−0.305; 0.038
ADMA					
Genus	*Lactococcus*	0.303; 0.038	Class	*Erysipelotrichia*	−0.326; 0.025
			Family	*Erysipelatoclostridiaceae*	−0.295; 0.046
			Genus	*Fournierella*	−0.375; 0.009
Big endothelin-1					
Genus	*Bilophila*	0.351; 0.020	Family	*Alcaligenaceae*	−0.314; 0.038
Genus	*Sutterella*	0.362; 0.016	Family	*Caulobacteraceae*	−0.327; 0.030
			Genus	*Achromobacter*	−0.299; 0.049
			Genus	*Alloprevotella*	−0.324; 0.032
			Genus	*Caulobacter*	−0.320; 0.030

**Table 6:** Significant differences in the relative abundance (%) of gut microbiota taxa between patients with high (fourth quartile) and low (first quartile) levels of endothelial dysfunction biomarkers.

Taxon	High Level	Low Level	*p*
Nitrate			
*Proteobacteria*	5.22 [3.87–13.9]	1.86 [1.28–4.90]	0.019
*Oscillospiraceae*	1.67 [0.73–2.80]	5.56 [1.96–9.09]	0.035
*Subdoligranulum*	0.67 [0.00–2.32]	2.94 [1.97–4.21]	0.017
*Rikenellaceae*	0.24 [0.00–2.27]	2.37 [0.85–3.36]	0.040
*Acidaminococcaceae*	0.00 [0.00–0.27]	0.77 [0.00–1.70]	0.035
*Christensenellaceae*	0.00 [0.00–0.01]	0.39 [0.03–1.06]	0.017
*Anaerovoracaceae*	0.00 [0.00–0.02]	0.15 [0.06–0.17]	0.011
*Erysipelatoclostridium*	0.09 [0.03–0.96]	0.00 [0.00–0.10]	0.040
*Peptococcaceae*	0.00 [0.00–0.00]	0.01 [0.00–0.34]	0.043
*Anaerotruncus*	0.00 [0.00–0.00]	0.01 [0.00–0.09]	0.023
*Paludicola*	0.00 [0.00–0.00]	0.00 [0.00–0.02]	0.033
ADMA			
*Erysipelotrichia*	0.62 [0.35–1.33]	2.20 [1.27–3.80]	0,008
*Erysipelatoclostridiaceae*	0.33 [0.23–0.55]	0.79 [0.26–1.73]	0.015
*Bilophila*	0.05 [0.01–0.11]	0.00 [0.00–0.03]	0.026
*Lactococcus*	0.01 [0.00–0.03]	0.00 [0.00–0.00]	0.048
*Fournierella*	0.00 [0.00–0.00]	0.00 [0.00–0.34]	0.016
*Dielma*	0.00 [0.00–0.01]	0.00 [0.00–0.00]	0.037
Big endothelin-1			
*Bilophila*	0.08 [0.01–0.14]	0.00 [0.00–0.03]	0.041
*Coprobacillus*	0.02 [0.00–0.16]	0.00 [0.00–0.00]	0.045
*Alloprevotella*	0.00 [0.00–0.00]	0.00 [0.00–0.51]	0.023
*Pedobacter*	0.00 [0.00–0.00]	0.00 [0.00–0.01]	0.007
*Sphingobacteriaceae*	0.00 [0.00–0.00]	0.00 [0.00–0.01]	0.019

## Data Availability

The datasets are available from the corresponding author upon reasonable request.

## Supplementary Materials

The following supporting information can be downloaded at: https://www.mdpi.com/article/10.3390/ijms25041988/s1.
