# Supplementary material for: Gut Microbiota and Biomarkers of Endothelial Dysfunction in Cirrhosis
Source: Int J Mol Sci. 2024 Feb 6;25(4):1988. doi: 10.3390/ijms25041988 (PMC10888218; doi:10.3390/ijms25041988)
Supplement: Supplementary file 1 [file ijms-25-01988-s001.zip › ijms-2823321-supplementary.pdf]

Supplementary Table S1. Characteristics of patients with cirrhosis

Age, years	49[44-56]
Body mass index, kg/m^2	26.1[24.5-30.0]
Male/female	18/29
Etiology of cirrhosis: alcohol	23 (48.9%)
HBV	3 (6.4%)
HCV	8 (17.0%)
metabolic-associated fatty liver disease	3 (6.4%)
mixed	7 (14.9)
cryptogenic	3 (6.4%)
Child–Pugh score	9[7-10]
End-diastolic volume of the left ventricle, mL	98[88-109]
Ejection fraction of the left ventricle, %	60[59-63]
Stroke volume, mL	59[55-66]
Heart rate, bpm	72[68-78]
Cardiac output, L/min	4.4[4.0-5.2]
Mean blood pressure, mmHg	87[80-93]
Systemic vascular resistance, dyn·s·cm^{-5}	1524[1302-1808]
Mean pulmonary artery pressure, mmHg	23[21-26]
Esophageal varices (Grade 1), n (%)	15 (31,9%)
Esophageal varices (Grade 2-3), n (%)	27 (57.4%)
Minimal hepatic encephalopathy, n (%)	32 (68.1%)
Overt hepatic encephalopathy, n (%)	4 (8.5%)
Ascites, n (%)	33 (70.2%)
Ascites (Grade 1), n (%)	22 (46.8%)
Ascites (Grade 2-3), n (%)	11 (23.4%)
Serum total protein, g/L	72.5[66.6-76.1]
Serum albumin, g/L	35.0[31.8-38.0]
Hypoalbuminemia (serum albumin < 35 g/L), n (%)	23 {48.9%)
Serum total bilirubin, μmol/L	45.3[29.2-64.5]
Hyperbilirubinemia (total bilirubin > 34 μmol/L), n (%)	32 (68,1%)
International normalized ratio (INR)	1.5[1.38-1.69]
Hypocoagulation (INR > 1.7), n (%)	10 (21.3%)
Fibrinogen, g/L	2.2[1.6-2.7]
Serum cholesterol, mmol/L	4.3[3.1-5.1]
Serum creatinine, mg/dL	74[66-97]
Serum sodium, mmol/L	141[140-142]
Serum potassium, mmol/L	4.3[4.0-4.8]
Serum glucose, mmol/L	4.8[4.3-5.5]
Alanine aminotransferase, U/L	31[21-44]
Aspartate aminotransferase, U/L	49[36-69]
Gamma glutamyl transferase, U/L	86[49-126]
Alkaline phosphatase, U/L	242[194-315]
C-reactive protein, mg/L	7[3-13]
Splenic length, cm	15.0[13.4-17.0]
